# Touchscreen typing-pattern analysis for detecting fine motor skills decline in early-stage Parkinson’s disease

**DOI:** 10.1038/s41598-018-25999-0

**Published:** 2018-05-16

**Authors:** Dimitrios Iakovakis, Stelios Hadjidimitriou, Vasileios Charisis, Sevasti Bostantzopoulou, Zoe Katsarou, Leontios J. Hadjileontiadis

**Affiliations:** 1 Department of Electrical and Computer Engineering, Aristotle University of Thessaloniki, Thessaloniki, Greece; 2 Third Neurological Clinic, G. Papanikolaou Hospital, Thessaloniki, Greece; 3 Department of Neurology, Hippokration Hospital, Thessaloniki, Greece; 4 Department of Electrical and Computer Engineering, Khalifa University of Science and Technology, Abu Dhabi, UAE

## Abstract

Parkinson’s disease (PD) is a degenerative movement disorder causing progressive disability that severely affects patients’ quality of life. While early treatment can produce significant benefits for patients, the mildness of many early signs combined with the lack of accessible high-frequency monitoring tools may delay clinical diagnosis. To meet this need, user interaction data from consumer technologies have recently been exploited towards unsupervised screening for PD symptoms in daily life. Similarly, this work proposes a method for detecting fine motor skills decline in early PD patients via analysis of patterns emerging from finger interaction with touchscreen smartphones during natural typing. Our approach relies on low-/higher-order statistical features of keystrokes timing and pressure variables, computed from short typing sessions. Features are fed into a two-stage multi-model classification pipeline that reaches a decision on the subject’s status (PD patient/control) by gradually fusing prediction probabilities obtained for individual typing sessions and keystroke variables. This method achieved an AUC = 0.92 and 0.82/0.81 sensitivity/specificity (matched groups of 18 early PD patients/15 controls) with discriminant features plausibly correlating with clinical scores of relevant PD motor symptoms. These findings suggest an improvement over similar approaches, thereby constituting a further step towards unobtrusive early PD detection from routine activities.

## Introduction

Parkinson’s disease (PD) is a common progressive neurodegenerative disorder^1^, characterised primarily by motor symptoms that contribute to significant disability^2,3^. The pathological hallmark of the disease is the loss of dopaminergic neurons in the substantia nigra, a basal ganglia structure of the human brain, and the presence of Lewy body-containing alpha-synuclein^3^, a protein widely distributed in the brain. The clinical spectrum of the disease is more extensive covering also a wide range of non-motor symptoms^4^ due to the degeneration of other dopaminergic and non-dopaminergic regions of the brain, spinal cord and peripheral nervous system^5–7^. The resultant decreased availability of dopamine in the basal ganglia leads to the motor symptomatology^3,8^. Due to the mildness of many early signs, including motor symptoms^2^, patients may not undergo clinical examinations for PD during early stages and therefore, the disease may be undiagnosed for many years^9^.

When PD is screened non-instrumentally - that is the majority of cases - the procedure traditionally involves the evaluation of subject’s overall condition according to standardised scales and questionnaires, such as the commonly used Unified Parkinson’s Disease Rating Scale (UPDRS)^10^. Motor status in particular, is often assessed by an expert based on the individual scoring and aggregated score of UPDRS Part III items^10^, which cover a broad range of PD motor symptoms, including among others, tremor (resting and action), rigidity, and bradykinesia. The latter examination, as is the case with other scales/questionnaires, requires a movement disorders specialist and the presence of the subject at the clinic. These factors limit the frequency of evaluation and monitoring of PD symptoms, while results are often of subjective nature as they rely on the expert’s experience or subject’s self-reports^11^. On the other hand, the development of more accessible tools that provide objective information with higher sampling rate can lead to timely diagnosis and consequent improvement of the prospective patient’s quality of life via early therapeutic interventions^12^.

To this end, data captured from electronic sensors have been used by works targeting objective PD symptoms monitoring, such as microphone-captured speech signals for voice impairment recognition^13^ and inertial measurement unit (IMU) data for hand tremor assessment^14^ or freezing of gait detection^15^, among others^16^, with real-life transferability potential. Moreover, the booming of mobile technology and user-mobile interaction^17^ has led to efforts of transferring PD screening and monitoring in the daily life, through mining of interaction data. The mPower study^18^ is the most prominent example of large-scale data collection for PD symptoms research, exploiting the deep penetration of smartphones in the general population. During the study, over 9,000 participants (PD patients and healthy users) remotely contributed multi-modal data by performing digitised tests bundled with a smartphone application. However, the drop-out rate concerning certain of these tests was over 90%. The latter highlights a common drawback of such approaches, i.e., data collection requires the active participation of the user and it is therefore, subject to adherence and impacted to a certain extent by the Hawthorne effect^19^.

In the modern era of computers and smartphones, typing on keyboards is a common, daily user-device interaction that involves dense (in terms of time), coordinated and successive finger/hand movements. Quantitative information arising from this type of interaction has been the focus of various applications, as it can be captured unobtrusively in the background during routine typing, reflecting in this way, the natural behaviour of the user. In particular, timing information associated with keystrokes, namely keystroke dynamics, has been exploited towards biometric authentication^20^, as well as Alzheimer’s disease^21^ and psycho-motor impairment detection, such as sleep inertia^22^. The fact that PD patients lack on rhythm stability of their finger movements^23,24^, while rigidity and bradykinesia affect their coordination^25^, renders keystroke dynamics, being the product of fine motor skills, an attractive source of information also for PD research. In fact, recent efforts have taken advantage of traditional keystroke dynamics timing variables, such as the hold time (HT) (the time a key is held down) and flight time (FT) (the time interval between releasing a key and pressing the next one), to classify subjects as having PD or not. Giancardo *et al*.^26^ used statistics of HTs, emerging from typing on a hardware keyboard, and Support Vector Machines (SVM) ensemble regression to produce a numerical index for distinguishing early PD patients and healthy controls with promising results [0.81 area under the ROC curve (AUC)]. To the same end, Arroyo-Gallego *et al*.^27^, based on data captured during typing on a touchscreen smartphone, evaluated the univariate and multivariate classification performance of various FT features and achieved a 0.91 AUC with a single feature. Both studies included tasks of continuous typing for more than five minutes.

Motivated by the aforementioned, the present work proposes a machine learning-based approach for discriminating early PD patients from healthy subjects based on enriched keystroke dynamics information, acquired during natural typing on a touchscreen smartphone. The dataset under scrutiny was recorded from 33 subjects (18 early PD patients/15 healthy controls) through a more ecologically-valid experiment compared to previous studies^26,27^ (see Dataset acquisition in Methods), which included fragmentary typing of short text excerpts, rather than continuous typing of longer duration. A systematic process is adopted to reach an optimised classification configuration by leveraging traditional variables of keystroke dynamics already exploited by relevant works^26,27^, such as HT and FT, which are further enriched for the first time, with information of normalised pressure (NP) applied on each keystroke/tap.

**Figure 1:**
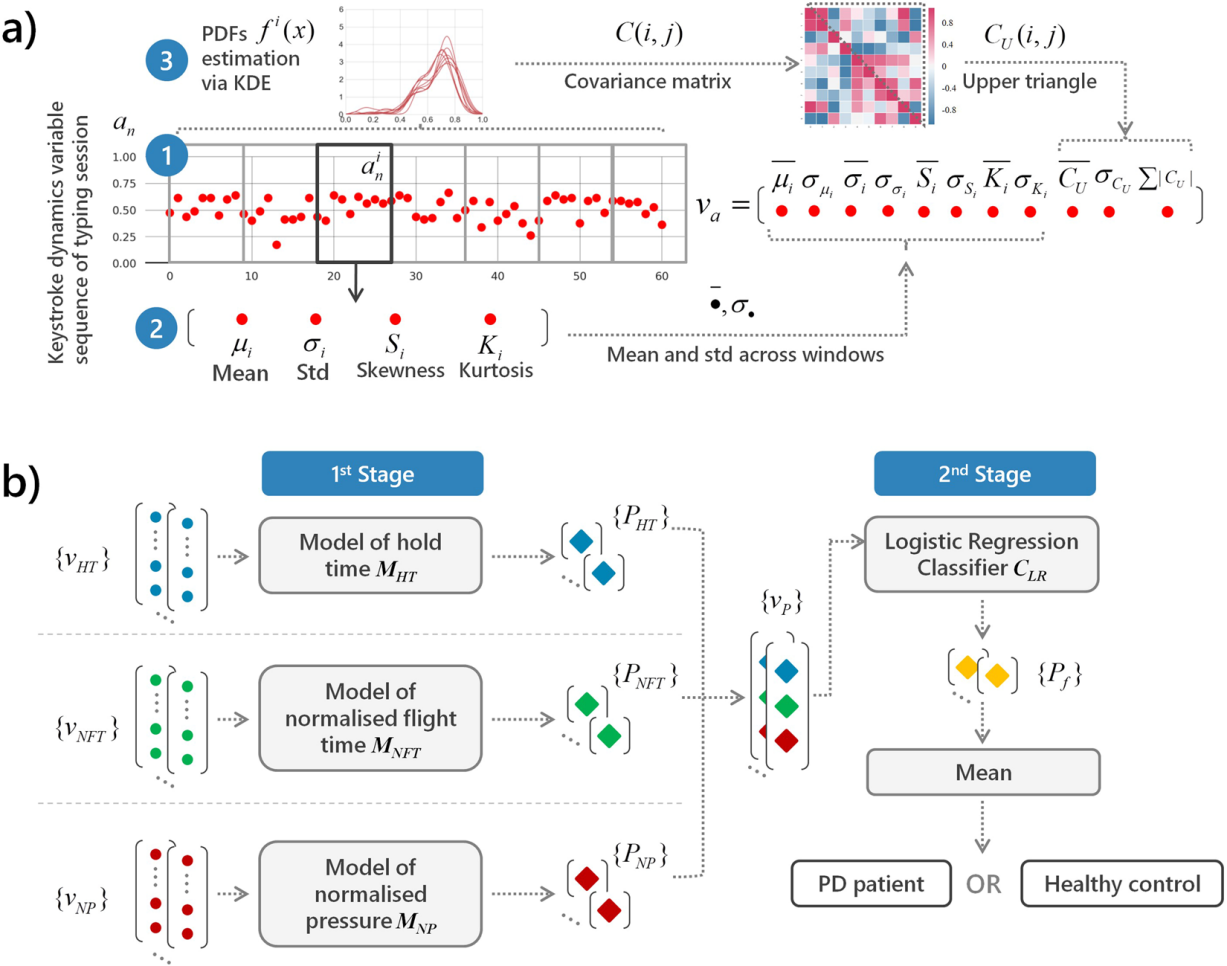
Illustration of (**a**) feature vector extraction from a given keystroke dynamics variable of a typing session and (**b**) classification pipeline of each subject based on hold time (HT), normalised flight time (NFT) and pressure (NP) information. (**a**) Given a keystroke dynamics variable sequence *a*_*n*_, *a* ∈ {*HT*, *NFT*, *NP*}: (1) The sequence is split in subsequences ${a}_{n}^{i}$ using 15-seconds non-overlapping time windows; (2) For each subsequence, the first- up to fourth-order statistical moments (mean *μ*_*i*_, standard deviation *σ*_*i*_, kurtosis *K*_*i*_, and skewness *S*_*i*_) of the elements are computed; (3) The probability density function (PDF) *f* ^*i*^(*x*) of each subsequence is estimated through kernel density estimation (KDE) and the matrix of sample covariance *C*(*i*, *j*) between the PDFs of all subsequences is calculated. Feature vectors *v*_*a*_ representing each typing session are formed by the mean $\mathop{\bullet }\limits^{\mbox{--}}$ and standard deviation (std) *σ*_⋅_ of the moments extracted in (2), across time windows (subsequences), and the mean, std and sum of absolute values of the upper triangle *C*_*U*_(*i*, *j*) of the covariance matrix calculated in (3). (**b**) The proposed two-stage multi-model pipeline for classifying subjects as PD patients or healthy controls: (1st Stage) Feature vector sets {*v*_*a*_} of a given subject, with each vector representing a typing session, serve as input to three trained models *M*_*a*_, each one dedicated to a keystroke dynamics variable, *a* ∈ {*NFT*, *HT*, *NP*}. Models *M*_*a*_ yield three prediction probabilities *P*_*a*_ which are then grouped in new feature vectors *v*_*P*_; (2nd Stage) Feature vector set {*v*_*P*_} serves as input to a Logistic Regression classifier *C*_*LR*_ that outputs the final classification probabilities {*P*_*f*_} denoting whether each typing session belongs to a PD patient or a healthy control. Finally, the mean of prediction probabilities *P*_*f*_ is used to categorise the subject as PD patient or healthy control.

Figure 1 illustrates the feature extraction process and the proposed classification pipeline. Regarding the former (Fig. 1(a)), features are extracted on a typing session level, by aggregating statistical characteristics of time-windowed keystroke dynamics variables to construct feature vectors representing the session on the HT, conditionally-filtered/normalised FT (NFT) and NP dimensions, individually. A leave-one-subject-out (LOSO) scheme with nested cross-validations is employed for feature selection, classifiers optimisation, training and testing (see Classification methodology in Methods) of the proposed approach. The latter is a two-stage multi-model pipeline (Fig. 1(b)), in the process of which three models are employed to classify each typing session of a given subject based on HT, NFT and NP feature vectors, independently (First stage), followed by a classifier that uses the individual outcomes to produce a fused probability on whether the session belongs to a PD patient or not (Second stage). In the end, prediction probabilities assigned to individual typing sessions are subjected to mean-voting to reach a final verdict on the subject’s status against PD. The performance of our method is evaluated using the receiver operating characteristics (ROC) analysis (see Classification performance evaluation in Methods) and compared against the most recent FT-based univariate and multivariate classification approaches^27^. The relationship of UPDRS Part III items of interest with the most discriminant features that emerge from the analysis is further examined to interpret the results.

**Figure 2:**
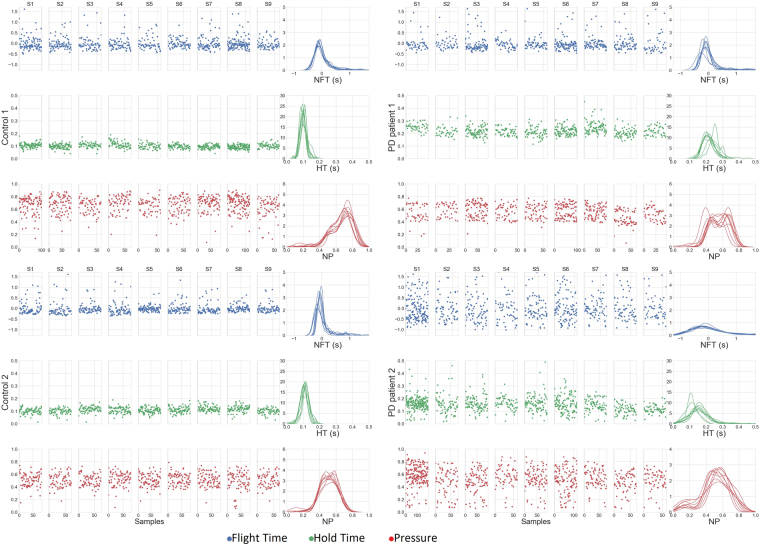
Indicative examples of keystroke dynamics variable sequences of healthy controls and PD patients. Sequences of normalised flight time (NFT) (blue), hold time (HT) (green), and normalised pressure (NP) (red) derived from 10 typing sessions (S1–S10), typed by two controls (Control 1 and Control 2) and two PD patients (PD Patient 1 and PD Patient 2), are presented along with overlayed probability density functions of each sequence, estimated for each typing session. Between two consecutive sessions, there was an one-minute interval. It is observed that controls exhibit similar behaviour across all variables. On the other hand, there are differentiations in the behaviour of PD patients when compared to each other, as well as healthy subjects. PD Patient 1 exhibits similar values to controls in terms of NFT and NP, but clearly higher HT values. In contrast, PD Patient 2 produced more wide-spread values for all keystroke dynamics variables in comparison to controls.

To exemplify the motivation behind the aforementioned classification approach, Fig. 2 depicts the HT, NFT, and NP sequences derived from 10 typing sessions of two healthy controls and two early PD patients that participated in our experiment. It can be observed that across sessions, all subjects exhibit a constant behaviour in terms of all keystroke dynamics variables. Nevertheless, while healthy subjects further exhibit similar behaviour across variables, PD patients present with differentiations when compared to each other and controls. In the light of this observation and after its generalisation, the two-stage multi-model approach was conceptualised. It, initially, granulates decisions to account for (non-)existing between-group differences in terms of each variable independently and afterwards, produces a final outcome on the basis of these individual decisions.

## Results

**Figure 3:**
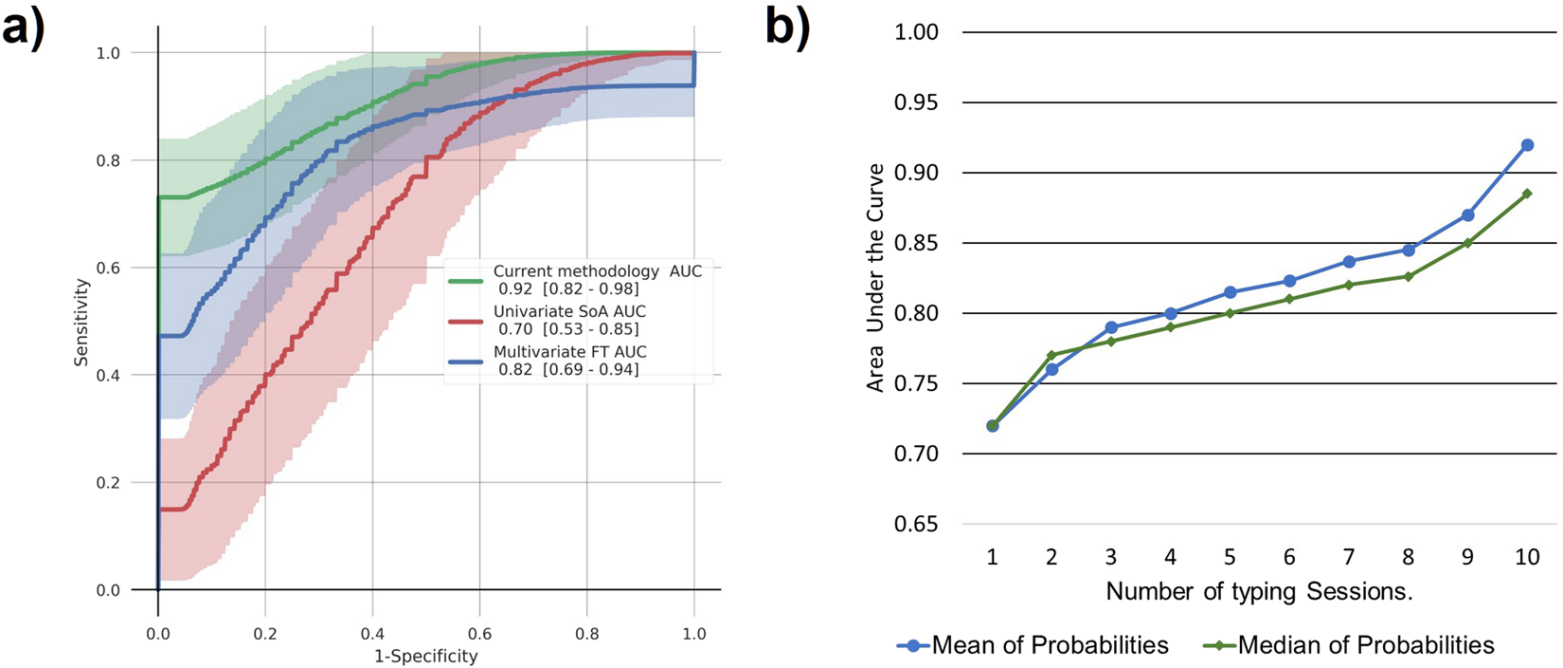
Comparison of Receiver Operating Characteristics (ROC) curves and evolution of area under the ROC curve (AUC) with respect to the number of typing sessions used as input. (**a**) ROC curves demonstrating the classification performance of the proposed approach and of existing ones reported in literature and applied on our dataset (18 early PD patients/15 controls). The performance of the proposed model (green curve) is compared to the best multivariate method based on flight time (FT) features proposed by Arroyo-Gallego *et al*.^27^ (blue curve), as well as their best performing univariate method (red curve). Solid lines represent the mean ROC curve, while shadowed areas delimit the 95% confidence intervals, computed over 1,000 bootstraps. The legend shows the AUC and the 95% confidence intervals. The two-stage multi-model approach described in this paper achieves an AUC of 0.92 [0.82–0.98], outperforming the univariate 0.70 [0.53–0.85] and multivariate 0.82 [0.69–0.94] methods^27^. (**b**) Evolution of the AUC for the best performing configuration of the proposed classification pipeline with respect to the number of typing sessions used as input. The blue and green line correspond to the AUC obtained by using the mean and median of final classification probabilities {*P*_*f*_}, respectively, as the voting scheme to reach a decision on whether the subject has PD or not based on her/his individually classified typing sessions (see also Fig. 1(b)). As intuitively expected, as the number of typing sessions increases, a clear improvement in the classification performance is noticed, while the mean of probabilities produces better results compared to the median value.

ROC-based performance comparisons between the best configuration of our classification pipeline and recent multivariate and univariate benchmark methods^27^ are presented in Fig. 3(a). The best configuration consisted of Ridge^28^ feature selection (during LOSO training) - Random Forest^29^ classifier combination for the three first-stage models and mean-voting as the final step for reaching a decision on the left-out subject (PD patient or control). With this configuration, an average AUC of 0.92 (0.82–0.98; 95% Confidence Interval (CI)) was achieved over 33 iterations of LOSO validation and 1,000 bootstraps. On the other hand, FT-based multivariate and univariate methods proposed by Arroyo-Gallego *et al*.^27^ had a lower performance, i.e., average AUC of 0.82 (0.73–0.94; 95%CI) and 0.70 (0.53–0.85; 95%CI), respectively, when applied on our dataset and subjected to the same LOSO validation process.

The diagnostic performance [AUC, diagnostic accuracy] of the proposed method that is based on fused probabilities {*P*_*f*_} was also compared against the individual performance of prediction probabilities estimated based on each keystroke dynamics variable, i.e., {*P*_*HT*_}, {*P*_*NFT*_}, {*P*_*NP*_} (see Fig. 1(b) and Supplementary Material Table S.3 for additional performance metrics). In this context, for each of the latter sets of probabilities, mean-voting was applied to reach a decision on the subject’s condition (PD patient or control). Results obtained for individual predictions ({*P*_*HT*_}: [82.1%, 78.8%]; {*P*_*NFT*_}: [77%, 66.7%]; {*P*_*NP*_}: [67%, 66.7%]), extracted by the best first-stage classification configuration, denote that by combining information of keystroke dynamics (fusion via the second stage), diagnostic performance is increased ({*P*_*f*_}: [92%, 82%]).

To mitigate the risk of over-fitting, the classification pipeline was trained in a subject-agnostic fashion, which allowed the use of a larger observation space (275 typing sessions) compared to 33 input observations if features were *a priori* aggregated on a subject level. The impact of the number of the left-out (test) subject’s typing sessions - feature representations of which are used as input - on classification performance is illustrated in Fig. 3(b). As intuitively expected, performance improves as information from a larger number of typing sessions becomes available. Figure 3(b) also provides a comparison of two voting-schemes, i.e., median and mean, used to reach the final decision on the subject’s condition, with the latter yielding higher AUC values compared to the former as the sample of final prediction probabilities becomes larger.

Table 1 presents the results of group-level (early PD patients vs controls) statistical comparisons (two-sided Mann-Whitney U test) with respect to each individual feature, as well as the frequency of selection of each feature over all LOSO iterations of the best classification configuration. From Table 1, it is evident that certain features differ significantly between groups and are consistently selected during the LOSO training step, thereby their discriminative power is highlighted. Such consistency in not observed when using benchmark^27^ FT feature extraction and multivariate LOSO analysis on our dataset (see Supplementary Material Table S.4).

**Table 1:** Results of statistical comparisons between the two groups (Early PD patients/Controls) for each extracted feature, along with the percentage of times the feature was selected during the leave-one-subject-out validation process.

Feature	NFT		HT		NP	
	Statistical Significance	Times selected (%)	Statistical Significance	Times Selected (%)	Statistical Significance	Times Selected (%)
$\overline{{\mu }_{i}}$	—	—	p < 0.001	**100%**	p < 0.001	**100%**
${\sigma }_{{\mu }_{i}}$	—	—	p < 0.001	3%	p < 0.001	0%
$\overline{{\sigma }_{i}}$	p < 0.001	0%	p < 0.001	**94%**	p = 0.013	0%
${\sigma }_{{\sigma }_{i}}$	p < 0.001	0%	p < 0.001	3%	p = 0.040	0%
$\overline{{S}_{i}}$	p < 0.001	**91%**	p = 0.010	0%	p < 0.001	0%
${\sigma }_{{S}_{i}}$	p = 0.136	0%	p < 0.001	0%	p < 0.001	0%
$\overline{{K}_{i}}$	p < 0.001	9%	p = 0.028	0%	p < 0.001	0%
${\sigma }_{{K}_{i}}$	p < 0.001	0%	p = 0.165	0%	p = 0.247	0%
$\overline{{C}_{U}}$	p = 0.248	0%	p < 0.001	0%	p = 0.468	0%
${\sigma }_{{C}_{U}}$	p = 0.002	0%	p < 0.001	0%	p < 0.001	0%
$\sum |{C}_{U}|$	p < 0.001	0%	p < 0.001	0%	p < 0.001	0%

Statistical significance is computed using the non-parametric two-sided Mann-Whitney U test. The percentage of times each feature is selected is computed over 33 loops of the leave-one-subject-out validation process for the best-performing configuration. Features selected consistently ($> \mathrm{90 \% }$ of times) are presented in bold and in all cases, they differ significantly between the two subject groups (p < 0.001). See Supplementary Material Table S.1 and Table S.2 for more detailed information on features.

**Figure 4:**
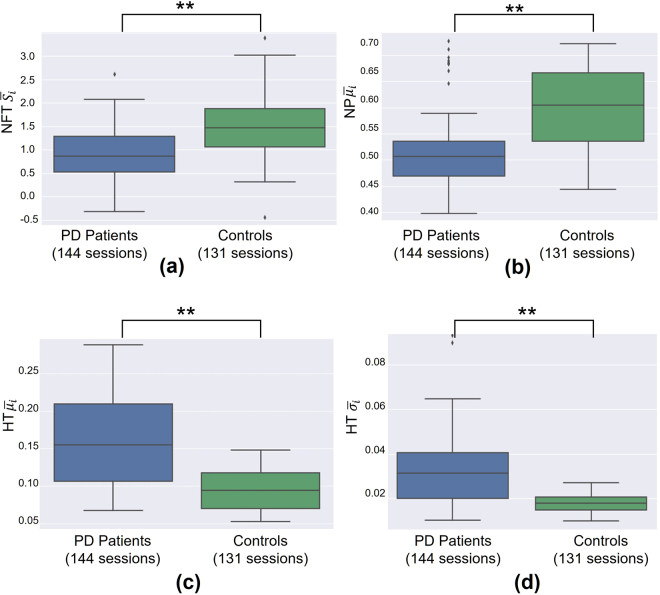
Group-wise comparison of distributions of the most frequently selected features as derived by the proposed approach. Box plots represent the distribution of HT $\overline{\mu i}$, HT $\overline{\sigma i}$, NFT $\overline{{S}_{i}}$, NP $\overline{\mu i}$ (see also Table 1) of PD patients and healthy controls, computed over 144 and 131 typing sessions, respectively. Each box plot visualises the interquartile range (height of rectangle), spanning the first (bottom) to the third quartile (top), the median value (horizontal line inside the rectangle), the minimum and maximum values (ends of “whiskers” below and above the box, respectively) still within the interquartile range, and outlier values (individual points below and above “whiskers”). The groups exhibit significant differences (two-sided Mann-Whitney U test) across all features of interest. **Statistically significant difference (p < 0.001).

Distributions of the most discriminant features per subject group, computed over all typing sessions, are illustrated in Fig. 4. In general, as compared to healthy controls, PD patients exhibit longer and more variant HTs, lower pressure values, and a shift towards longer FTs, as indicated by lower skewness values of the zero-mean NFT distributions. These relationships still hold even when the group of early PD patients is clustered and examined with respect to PD medication (Supplementary Material Fig. S.1). De-novo patients’ (recently diagnosed with PD and never taken PD medication) data form an intermediate distribution between healthy controls and early PD patients under medication. Both early PD patients’ subgroups differ significantly from controls in terms of feature distributions, but not when compared to each other. These findings are similar to those that Giancardo *et al*.^26^ reported regarding their proposed HT-based discriminant index.

To foster an interpretation of our outcomes with respect to PD motor symptomatology, results of Spearman correlation analysis between the most frequently selected features (see also Table 1) and scores of UPDRS Part III items of interest, as well as the compound score, are tabulated in Table 2. High to moderate correlation coefficients ($|{r}_{s}| > 0.60$, *p* < 0.001) were obtained for scores of rigidity and finger taps (right hand), as well as body bradykinesia/hypokinesia and the general motor status (total UPDRS Part III score). These results point to an effect of the severity of the associated motor symptoms on keystroke dynamics and consequently, typing kinetics. On the other hand, scores associated with the upper-left extremity, as well as bilateral action tremor scores, yielded low and in certain cases, insignificant correlations ($p > 0.05$). The fact that all subjects of the study cohort were right-handed may explain the stronger unilateral (right side) correlations observed for all relevant clinical scores.

**Table 2:** Results of correlation analysis between the most frequently selected features and UPDRS Part III single-item scores of interest/total score.

Selected Feature	UPDRS Part III Single Items							Total UPDRS Part III Score
	Finger Tapping (RH)	Finger Tapping (LH)	Rigidity UE (RH)	Rigidity UE (LH)	Action Tremor (RH)	Action Tremor (LH)	BK/HK	
HT $\overline{\mu i}$	0.60^†^	0.33^†^	0.69^†^	0.30^†^	0.39^†^	0.05^*n*.*s*.^	0.50^†^	0.53^†^
HT $\overline{\sigma i}$	0.64^†^	0.49^†^	0.71^†^	0.47^†^	0.42^†^	0.16^*n*.*s*.^	0.63^†^	0.62^†^
NFT $\overline{{S}_{i}}$	−0.48^†^	−0.46^†^	−0.51^†^	−0.45^†^	−0.35^†^	−0.11^*n*.*s*^	−0.58^†^	−0.51^†^
NP $\overline{\mu i}$	−0.60^†^	−0.56^†^	−0.55^†^	−0.52^†^	−0.40^†^	−0.31^†^	−0.62^†^	−0.62^†^

Values of Spearman’s correlation coefficient *r*_*s*_ are presented after correlating the most frequently selected features (HT $\overline{\mu i}$, HT $\overline{\sigma i}$, NFT $\overline{Si}$, NP $\overline{\mu i}$) (see also Table 1) with UPDRS Part III single-item scores of relevant PD motor symptoms, i.e., rigidity of upper extremities, action tremor, and bradykinesia (finger tapping and body bradykinesia-hypokinesia), as well as with the total UPDRS Part III score, across all subjects (both early PD patients and controls). All healthy controls were assigned a value of zero for each UPDRS Part III item. As the latter renders each UPDRS item variable skewed, Spearman’s rank correlation was employed for this analysis. BK/HK: Bradykinesia/Hypokinesia; RH: Right Hand; LH: Left Hand; UE: Upper Extremity; ^†^p < 0.001; n.s.: p > 0.05.

## Discussion

Digital transformation has the potential to deliver quantitative tools and personalised solutions to satisfy unmet societal health care needs. High-frequency, easily-accessible monitoring means can allow for availability of behavioural and biometric data that in turn, when analysed properly and combined with standard medical practices, can pave the way for the prognosis of diseases in early stages, with consequent benefits for patients. That is the case of PD, i.e., a disorder that is usually left undiagnosed for years due to the mildness of many early symptoms, while early treatment can significantly improve patients’ quality of life over the course of the disease. For this reason, PD detection and monitoring based on objective data arising from user-mobile interaction has been in the spotlight during recent years. The ability of unobtrusive capturing of such data further enhances their objective character as the information reflects the user’s natural behaviour and in addition, helps overcome issues of adherence that plague approaches requiring users’ active participation. Our method attempted to amalgamate all these desired parameters towards our aspiration to develop a tool for early PD screening in daily life. Results are rather promising, as classification performance denotes satisfactory discrimination between early PD patients and controls, based on features with plausible correlations with clinical scores of relevant symptoms.

In this study, data from a PD patients’ and a healthy controls’ group, matched in terms of demographics (see Table 3), were acquired and analysed. Data acquisition was based on a protocol emulating real-life conditions that included fragmentary typing of short text excerpts, which is common in daily user-mobile interaction, rather than continuous typing of longer duration ($$ > 5$$ min) that was employed in similar studies^26,27^. Resting intervals between short typing sessions also contributed towards the minimisation of fatigue effects on typing kinetics that could be otherwise amplified due to a continuous typing effort. Regarding the 18 early PD patients involved (Table 3), four of them have never received PD medication (de-novo) and all of them were at early stages (Hoehn-Yahr stages I or II, mean UPDRS Part III score/std 16.9/7.8) and recently diagnosed (mean disease onset yrs./std 2.5/1.6). These clinical characteristics of the study cohort strengthen the significance of the study outcomes, as the proposed method satisfactorily differentiates healthy controls from PD patients even at early stages of the disease.

**Table 3:** Summary of complete study cohort (33 subjects) demographic and clinical characteristics with respect to each group (Early PD patients and Controls).

	Early PD patients	Controls	Statistical Significance
n (total n = 33)	18	15	N.A.
**Demographics**			
Women # (%)	4 (22%)	7 (46%)	n.s. (p = 0.24)
Men # (%)	14 (78%)	8 (54%)	n.s. (p = 0.24)
Avg. Age, years (std)	61 (8.4)	57 (3.9)	n.s. (p = 0.67)
Subjects #/# who completed Education Level H/U	4/14	1/14	n.s. (p = 0.31)
Avg. Years of Smartphone Usage (std)	3.4 (1.6)	2.8 (2.6)	n.s. (p = 0.15)
**Clinical characteristics**			
Avg. Disease onset, years (std)	2.5 (1.6)	N.A.	N.A.
Avg. UPDRS Part III score (std)	16.9 (7.8)	0.0 (0.0)	sig. (p < 0.001)
PD patients #/# under treatment/De-novo	14/4	N.A.	N.A.
PD patients #/# with right/left most affected side	13/5	N.A.	N.A.
Subjects #/#/# with dominant hand Right/Left/Ambidextrous	18/0/0	15/0/0	N.A.
Avg. LEDD^a^, mg (std)	247 (110)	N.A.	N.A.

With the exception of clinical characteristics (UPDRS Part III score), the two groups are reasonably matched in terms of demographics as no significant differences (p < 0.05) are observed (two-sided Mann-Whitney U test). ^a^Avg. Levodopa Equivalent Daily Dose (LEDD) concerns only PD patients under treatment (n = 14). N.A.: not applicable; sig.: significant; n.s.: non-significant.

Furthermore, this work explored for the first time the combined discriminative potential of enriched keystroke variables (associated with both timing and pressure), unlike previous research efforts that focused on a sole dimension of traditional keystroke dynamics, i.e., the HT^26^ or FT^27^. Based on this combinatory approach, the best classification performance achieved here (0.92 AUC, 0.82/0.81 sensitivity/specificity) suggests an improvement over benchmark methods^27^ applied on our dataset (Fig. 3(a)), as well as on single keystroke variable-based variants of our method (Supplementary Material Table S.3). Moreover, the adopted classification pipeline brought to light statistical features of keystroke dynamics (HT $\overline{\mu i}$, HT $\overline{\sigma i}$, NFT $\overline{{S}_{i}}$, NP $\overline{\mu i}$) that differ significantly between subject groups and are in addition, consistently selected during LOSO training (Table 1). The discriminative power of these features is complemented by their explainable significant correlations with clinical scores (UPDRS Part III items) of PD motor symptoms (Table 2) that may affect typing kinetics, i.e., muscle rigidity and bradykinesia - hypokinesia. We further discuss these results in detail below as they provide evidence on the internal validity of our approach.

Overall, as all study participants were right-handed and they mainly used their dominant hand (alone or in combination with the left hand) to type during the experiment, correlations obtained for right-hand UPDRS Part III scores, where applicable, were consistently higher than those produced for the left extremity. Probing further, all discriminant features significantly differ between the two subject groups (see Fig. 4) and exhibit significant correlations (0.51 ≤ |*r*_*s*_| ≤ 0.62) with the total UPDRS Part III score that reflects the subject’s overall motor status. However, as the compound score includes evaluations of motor symptoms that are unlikely to affect typing kinetics, e.g., speech and facial expression, focusing on correlations with scores of more relevant symptoms (finger tapping, rigidity and action tremor of upper extremities, and body bradykinesia/hypokinesia) would provide more solid conclusions. For instance, and according to our results, action tremor appears to have moderate to low impact on typing kinetics, as correlation values of all discriminant features with the associated UPDRS Part III single-item score were generally low (|*r*_*s*_| ≤ 0.4) and in certain cases, insignificant ($p > 0.05$). This was the case for correlations of left hand action tremor with all features, except for the NP $\overline{\mu i}$, which may denote a minor effect of this symptom on pressure values.

On the other hand, as derived from distributions of the most discriminant HT features (HT $\overline{\mu i}$, HT $\overline{\sigma i}$), PD patients produced significantly longer (HT $\overline{\mu i}$ mean/std: PD 0.15/0.06 vs. controls 0.09/0.03) and more variant HTs (HT $\overline{\sigma i}$ mean/std: PD 0.03/0.01 vs. controls 0.02/0.00) compared to controls. These results might constitute a projection of the effects of rigidity (muscle stiffness) and bradykinesia (slowness of movement), as denoted by the moderate to high positive correlations (0.50 ≤ *r*_*s*_ ≤ 0.71) of these HT features with corresponding UPDRS Part III single-item scores (bradykinesia/hypokinesia and right-hand finger tapping, rigidity). These motor symptoms may slow down finger reflexes causing PD patients to hold down keys for longer and inconsistent time intervals. Rigidity, and especially bradykinesia, may also have an impact on the latency between keystrokes, as derived by significant negative correlations of the skewness-related FT feature (NFT $\overline{{S}_{i}}$) with corresponding UPDRS Part III single-item scores, i.e., bradykinesia/hypokinesia (*r*_*s*_ = −0.58) and right/left-hand rigidity (*r*_*s*_ = −0.51/*r*_*s*_ = −0.45). PD patients exhibited on average lower values of NFT distributions skewness across typing sessions as compared to controls (NFT $\overline{{S}_{i}}$ mean/std: PD 0.90/0.53 vs. controls 1.51/0.61). Provided that the NFT distributions of both subject groups are of zero-mean, due to normalisation of FT values, lower skewness denotes a shift of the mass of patients’ NFT distributions towards higher values (see also Fig. 2 for indicative examples); this indicates the existence of longer latencies between keystrokes during typing, possibly due to slower movements. In our case, PD patients and controls also exhibit significant differences in terms of pressure applied to initiate keystrokes, as denoted by the NP feature distributions (NP $\overline{\mu i}$ mean/std: PD 0.51/0.06 vs. controls 0.60/0.08). Significant negative correlations of this feature with right/left-hand finger tapping (*r*_*s*_ = −0.60/*r*_*s*_ = −0.56) and body bradykinesia/hypokinesia (*r*_*s*_ = −0.62) scores indicate that, as the severity of the associated symptom increases, pressure applied on touchscreen keys decreases on average. The latter could be attributed to inadequately-scaled movements (in terms of speed and amplitude) that constitute manifestations of hypokinesia that is present in PD^23^. On a side note, unilateral upper extremities rigidity (*r*_*s*_ = −0.55/*r*_*s*_ = −0.52 right/left hand) and action tremor (*r*_*s*_ = −0.40/*r*_*s*_ = −0.31 right/left hand) appear to have moderate to low projection on average pressure values, respectively.

One possible limitation of our study is that patients under dopaminergic therapy were asked to refrain from taking their medication at least eight hours before their morning visit to participate in the experiment. This time interval might not have been sufficient enough for certain patients to be in the “practically off” condition, a transition that usually requires 12 hours after the last dose^30^. The latter, combined with potential effects of long-duration response to Levodopa^31,32^, may have improved the psychomotor state of these patients and consequently, their typing cadence, leading to a reduced discrimination performance across classification methods tested. From an overall perspective however, the best configuration of our approach exhibits a very promising potential in correctly classifying early PD patients and controls (average AUC = 0.92), despite any “echoing” effects of dopaminergic therapy on certain study participants’ fine motor skills.

Since the proposed approach is based on the analysis of quantitative data that can be collected in an unobtrusive fashion during an activity of daily living, privacy issues should be considered. In fact, our method is privacy-aware, as it is based on timing and pressure variables of keystrokes, without requiring the actual content of the typed text. Nevertheless, results of this work, as well as of similar research efforts, indicate that this source of information can provide insights into users’ health status and therefore, it belongs, by definition, to the category of sensitive personal data. The ability to unobtrusively integrate the recording of typing patterns with routine activities highlights even more the need to ensure that users are aware in advance of what data will be recorded, when and to which end, before using relevant applications. In this context, future commercial applications exploiting such patterns towards the recognition of psycho-motor impairments must comply with ethical guidelines and data protection regulations, as per standard practices in similar cases involving recordings of biometrics and health data.

In summary, our work provided evidence on a machine-learning method that satisfactorily detected early-stage PD in a relatively small cohort, based on the projection of fine-motor skills decline on keystroke dynamics variables during natural typing. Correlation results indicate the potential of evolving the binary classification problem into a regression analysis for estimating the severity of individual PD motor symptoms based on relevant statistical features of keystroke variables. A future deployment of the latter could assist physicians to gain objective, granular and explainable insights into the subject’s condition against PD. Beyond this study, our vision is to extend and validate the developed method on longitudinal data, unobtrusively captured from a large pool of subjects and their day-to-day typing interaction with their smartphone. This vision is consequent to the overarching aim to develop accessible, non-intrusive tools for early PD screening in daily life of the European Horizon 2020 project “i-PROGNOSIS” (www.i-prognosis.eu), within the framework of which the present work was realised.

## Methods

A feature extraction and classification pipeline is presented for classifying subjects as PD patients or healthy controls based on data derived from sporadic typing sessions on a touchscreen smartphone. The data of interest comprise sequences of hold times (HT) (the time between pressing and releasing a key) and flight times (FT) (the time between releasing a key and pressing the next one), produced from captured time stamps, as well as normalised pressure (NP) sequences. Each typing session is characterised via three independent feature vectors, each composed of lower-order (mean, standard deviation) and higher-order (kurtosis, skewness, and covariance) statistics representing the HT, FT and NP sequences, respectively. For each typing session, a two-stage multi-model classification scheme is adopted with the first stage yielding classification probabilities based on each of the feature vectors of HT, FT, and NP sequences and the second stage fusing these probabilities to produce a final prediction probability for the particular session (belonging to a PD patient or a healthy control). The mean of the probability distribution formed by classifying all typing sessions of a subject is used to reach the final decision on her/his condition (PD patient or healthy control).

### Study procedures

The study protocol was approved by the Aristotle University of Thessaloniki, Greece (Bioethics Committee of Medical School, approval no. 359/3.4.17). Informed consent was obtained from all subjects prior to their participation in the study. Subjects held the right to withdraw from the procedure at any time, without providing any justification. Recruitment and study procedures were carried out according to institutional and international guidelines on research involving adult human beings.

### Study cohort

The study cohort comprised two groups, i.e., the early PD patients’ group that consisted of 18 subjects (Hoehn-Yahr stages I or II) with a confirmed diagnosis of less than five years and the controls’ group that included 15 healthy subjects without any sign of Parkinsonism. The two groups were matched in terms of gender, age, education level, and years of experience with smartphones. All subjects were native Greek speakers, 40 years of age or older, and right-handed. Demographic and clinical characteristics of the study cohort are tabulated in Table 3. Only participants who self-reported that they used a touchscreen-equipped smartphone for at least a year were considered eligible. Subjects with other diagnosed psycho-motor impairments (including drug abuse and sleep disorders), cognitive dysfunctions, upper limb functional limitations (including recoveries from recent surgery) or uncorrected vision problems were excluded from the study. Subjects were recruited from two neurological clinics in Thessaloniki (Greece) and the Aristotle University of Thessaloniki (Greece). The study protocol (typing experiment and clinical evaluation) was conducted during a single morning visit of each subject at one of the clinics. Patients receiving symptomatic relief medication for PD were asked to refrain from taking it for at least eight hours before their visit, i.e., they practically underwent all study procedures in the “off-state”, before their morning dose.

### Dataset acquisition

Each visit included a typing experiment and a clinical evaluation. During the typing experiment, subjects were asked to transcribe 11 text excerpts of the famous fairy-tale “The Little Prince” in Greek, using an Android smartphone (LG Nexus 5X with a screen of 5.2 inches in diagonal and a resolution of 1080 × 1920 pixels, running native Android 7.0) and a custom mobile application with an editable text placeholder. The text excerpts were presented on a 17-inch laptop in front of the subject. The first excerpt was 200 characters-long and it was the same for all subjects, in order to familiarise themselves with the software keyboard and the mobile device. Data acquired during this session were not included in the analysis. Following this, each subject typed 10 short text excerpts (46–115 characters-long), pseudo-randomly and uniformly drawn from the fairy-tale, with an one minute-interval between two consecutive excerpts. Subjects were instructed to sit comfortably and to type using their own typing style (one or both hands) and capital letters only. There were no time constraints for typing the excerpts and participants were at liberty to adjust their posture and to correct typing errors or not. The goal was to simulate real-life, natural typing interaction with mobile devices, i.e., sporadic short typing sessions during the day, so as to acquire a dataset as ecologically-valid as possible in a laboratory environment. A custom software keyboard was developed for the Android Operating System (OS) to capture the raw data of interest in the background, while typing. These included the raw time stamps of the press and release touch events for each key tapped (in milliseconds) and the normalised pressure (0.000–1.000) applied on each key tap, as outputted by native functions of the OS; raw pressure values are not exposed by the OS. The captured time stamps and pressure values were recorded in a separate .txt file for each typing session, stored on the device internal storage memory. The filename of each file included the coded ID assigned to the subject at the beginning of the experiment. The keyboard did not support any “long press” actions that would constitute noise artefacts. Nevertheless, in a more advanced keyboard that supports such actions, relevant key presses can be easily flagged and excluded from a subsequent analysis.

Regarding clinical evaluation, after completing the typing experiment, each participant was subjected once to evaluation in terms of the motor section of the Unified Parkinson’s Disease Rating Scale (UPDRS-Part III)^10^ that was performed by a specialised neurologist. UPDRS-Part III scores were afterwards logged, along with demographic data, in a spreadsheet file and mapped to the subjects’ coded IDs by the neurologist.

### Feature vector extraction

Let ${t}_{n}^{p}$ and ${t}_{n}^{r}$ be monotonically increasing sequences of time stamps (ms) corresponding to the press (*p*) and release (*r*) of key n = *1*, *2*, …, *N*, respectively, where *N* is the total number of keys pressed during a typing session. A lower bound of 20 characters per minute is set for the typing rate per typing session; sessions which did not meet this bound were omitted from the subsequent analysis. The sequences of HTs and FTs are defined as $H{T}_{n}={t}_{n}^{r}-{t}_{n}^{p}$, n = *1*, *2*, …, *N*, and $F{T}_{n}={t}_{n+1}^{p}-{t}_{n}^{r}$, n = *1*, *2*, …, *N-1*, respectively, and the normalised pressure sequence as $N{P}_{n}=NP({t}_{n}^{p})$, n = *1*, *2*, …, *N*, where *NP*(*t*) is the varying normalised pressure applied during the pressing of each key.

To further minimize the effects of varying typing dexterity amongst subjects and remove outlier values (due to subjects stalling key presses because of reading the text to be transcribed on the laptop screen), conditional filtering is applied to the FT sequence which is affected the most by these factors. The filtering approach^27^ of Arroyo-Gallego *et al*. was adopted here for the analysis results to be comparable. FT values exceeding a 3 s-threshold are removed from the sequence, *FT*_*n*_ sequences are further detrended and values outside the 99% interval of a real-life data distribution [−1.27,1.7] (s) are filtered out. The latter real-life data distribution was estimated by leveraging an external dataset of ten healthy participants who used our Android custom keyboard for typing in their daily life for more than three weeks. Thus, the conditionally-filtered *FT*_*n*_ sequence for each typing session, hereby referred to as the normalised flight time sequence *NFT*_*n*_, can be written as:

1 $$NF{T}_{n}=F{T}_{n}-\overline{F{T}_{n}},NF{T}_{n}\in [\,-\,\mathrm{1.27,}\,\mathrm{1.7]}s,\,n=\mathrm{1,}\,\mathrm{2,}\,\mathrm{...,}\,N-1.$$

On the other hand, HT values (usually in the range of 100 milliseconds), being the relatively short timing outcome of a finger reflex - the finger presses down (to initiate the action) and releases the key (upon success of the intended action) after visual or haptic feedback that the key was registered - are not expected to be affected by the aforementioned collateral factors. For example, HT, unlike FT, is almost unaffected by interruptions during typing. Non-pathological factors that affect HT values, such as the type of medium (hardware with key travel or touch-based virtual keyboard), deliberate long-presses or the type of key feedback, were mitigated by the experiment protocol as no long-presses were supported and the virtual keyboard and key feedback (visual) was common among all subjects. In this context, no conditional filtering/normalisation is applied on the HT sequence, on par with works that exploited features of raw HTs for psycho-motor impairment detection^22,26^.

Thereafter, we define subsequences of *HT*_*n*_, *NFT*_*n*_, *NP*_*n*_ based on a time window *w* with duration *T* (*T* = 15 s in our implementation as in Arroyo-Gallego *et al*.^27^) as:

2 $$\begin{array}{ccc}H{T}_{k}^{i} & = & H{T}_{{n}_{k}},{n}_{k}={n}_{1},{n}_{2},\,\mathrm{...,}\,{n}_{w},{t}_{{n}_{1}}^{p} > (i-\mathrm{1)}T,{t}_{{n}_{w}}^{r} < iT,\\ N{P}_{k}^{i} & = & N{P}_{{n}_{k}},{n}_{k}={n}_{1},{n}_{2},\,\mathrm{...,}\,{n}_{w},{t}_{{n}_{1}}^{p} > (i-\mathrm{1)}T,{t}_{{n}_{w}}^{p} < iT,\\ NF{T}_{k}^{i} & = & NF{T}_{{n}_{k}},{n}_{k}={n}_{1},{n}_{2},\,\mathrm{...,}\,{n}_{w},{t}_{{n}_{1}}^{r} > (i-\mathrm{1)}T,{t}_{{n}_{w}}^{p} < iT,\end{array}$$

where *i* denotes the increasing index of the window and *k* = 1, 2, ..., *n*_*w*_ − *n*_1_ + 1 the index of the elements in the window. A minimum of five elements per subsequence (*n*_*w*_ − *n*_1_ >4) is considered in order to extract meaningful statistical features; subsequences not meeting this criterion were omitted from further analysis. For simplicity, we denote any of the valid subsequences as ${a}_{k}^{i}$. Statistical features extracted to represent the *i*-th subsequence are: the mean ${\mu }_{i}={\sum }_{k}{a}_{k}^{i}/({n}_{w}-{n}_{1}+\mathrm{1)}$, standard deviation ${\sigma }_{i}=\sqrt{\sum k({a}_{k}^{i}-{\mu }_{i})/({n}_{w}-{n}_{1})}$, skewness ${S}_{i}={\sum }_{k}{(\frac{{a}_{k}^{i}-{\mu }_{i}}{{\sigma }_{i}})}^{3}/({n}_{w}-{n}_{1}+\mathrm{1)}$ and kurtosis ${K}_{i}={\sum }_{k}{(\frac{{a}_{k}^{i}-{\mu }_{i}}{{\sigma }_{i}})}^{4}/({n}_{w}-{n}_{1}+\mathrm{1)}$ of the samples of the subsequence. Moreover, an approximation of the probability density function (PDF) *f* ^*i*^(*X*) of the *i*-th subsequence is estimated by employing Kernel Density Estimation (KDE)^22^ with a Gaussian kernel. In the present implementation, ten quantization levels (*L* = 10) are used and the bandwidth parameter (*b*) of the Gaussian kernels is calculated by applying the Sheather-Jones method^33^ on the aforementioned external dataset, resulting in *b* = 0.0060, 0.0289, and 0.0300 for HT, NFT, and NP data respectively. For each typing session, we construct three feature vectors, each representing a type of sequence (*HT*_*n*_, *FT*_*n*_ or *NP*_*n*_), by aggregating the statistical representations of all subsequences as:

3 $${v}_{a}={[\overline{{\mu }_{i}},{\sigma }_{{\mu }_{i}},\overline{{\sigma }_{i}},{\sigma }_{{\sigma }_{i}},\overline{{S}_{i}},{\sigma }_{{S}_{i}},\overline{{K}_{i}},{\sigma }_{{K}_{i}},\overline{{C}_{U}},{\sigma }_{{C}_{U}},\sum |{C}_{U}|]}_{a},$$

where *a*∈{*HT*, *NFT*, *NP*} and $\mathop{\bullet }\limits^{\mbox{--}},{\sigma }_{\cdot }$ denote the mean and the standard deviation of the corresponding values, respectively. *C*_*U*_ is the upper triangle of the covariance matrix formed by the values of sample covariance between the estimated PDFs *f* ^*i*^(*X*) of all subsequences, i.e., ${C}_{i,j}=\frac{1}{L-1}{\sum }_{X}[\,{f}^{i}(X)-\overline{{f}^{i}(X)}\,][{f}^{j}(X)-\overline{{f}^{j}(X)}]$, and $\sum |{C}_{U}|$ is the sum of absolute values of *C*_*U*_. In the case of flight time, $\overline{{\mu }_{i}}$ and ${\sigma }_{{\mu }_{i}}$ are omitted as *NFT*_*n*_ is of zero mean due to the aforementioned conditional filtering. After applying the feature vector extraction approach on all typing sessions from all subjects, three feature vector sets are produced, i.e., *I*_*HT*_ = {*v*_*HT*_}, *I*_*NFT*_ = {*v*_*NFT*_}, and *I*_*NP*_ = {*v*_*NP*_}.

### Classification methodology

**Figure 5:**
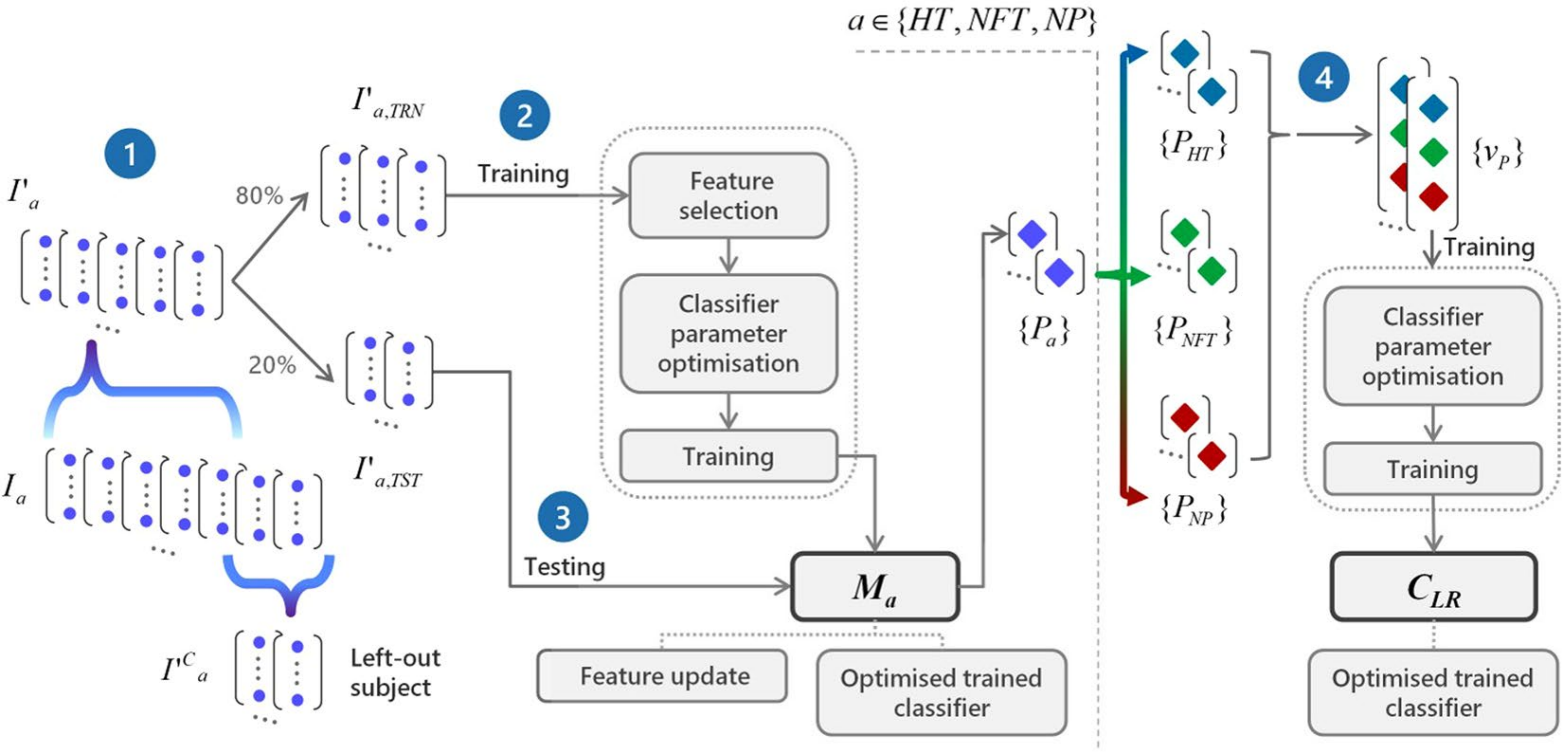
Training procedure of each leave-one-subject-out loop. (1) The computed *I*_*a*_ feature vector sets, *a*∈{*HT*,*NFT*,*NP*}, with each feature vector representing a typing session, are split in two parts, i.e., *I*′_*a*_ and ${I^{\prime} }_{a}^{C}$ (the left-out subject feature vector sets). The *I*′_*a*_ sets are further split in two subsets, i.e., the *I*′_*a*,*TRN*_ (80%) and the *I*′_*a*,*TST*_ (20%). (2) The *I*′_*a*,*TRN*_ subsets are used for feature selection, hyper parameter optimisation using an inner 4-fold cross-validation, and training of three classifiers, resulting in three independent models *M*_*a*_, each one dedicated to a specific keystroke dynamics variable (HT, NFT and NP). (3) The *I*′_*a*,*TST*_ subsets are then used to test the three *M*_*a*_ models, resulting in three classification probabilities sets {*P*_*a*_}, *a*∈{*HT*,*NFT*,*NP*}. (4) Sets {*P*_*HT*_}, {*P*_*NFT*_}, and {*P*_*NP*_} are fused in single feature vectors, forming a set {*v*_*P*_} that serves to optimise (as in (2)) and train a Logistic Regression classifier *C*_*LR*_. The end product of the training procedure is a configuration of three optimised/trained models (first stage) that independently classify each typing session according to each keystroke dynamics variable, and an optimised/trained Logistic Regression classifier (second stage) that aggregates the latter decisions to reach a final verdict on whether the typing session belongs to a PD patient or not. The latter two-stage configuration is tested using the left-out subject’s ${I^{\prime} }_{a}^{C}$ sets (see also Fig. 1(b)).

A two-stage classification pipeline is adopted for classifying subjects as having PD or not. In the first stage, three models (feature selection and classifier combination) are employed, with each one learning and predicting based on the representations of HT (*v*_*HT*_), NFT (*v*_*NFT*_), and NP (*v*_*NP*_) of each typing session, respectively, and the labels inherited from the corresponding subject’s condition (PD patient or healthy control). This approach was conceptualised after observing differences in each of these keystroke dynamics variables amongst PD patients (see Fig. 2). In the second stage, prediction probabilities produced by each of the three first-stage models are combined into a single vector (*v*_*p*_) and serve as input to a single classifier that yields a fused prediction probability on whether or not the particular typing session belongs to a PD patient or a healthy control. A leave-one-subject-out (LOSO) scheme with inner k-fold cross-validations is employed to evaluate the discrimination potential of the proposed approach. The training and testing procedures of the classification approach are illustrated in Figs. 5 and 1(b), respectively.

For the training step of each LOSO loop, each of the *I*′_*HT*_, *I*′_*NFT*_, *I*′_*NP*_ feature vector sets, derived from the complete *I*_*HT*_, *I*_*NFT*_, *I*_*NP*_ sets by leaving out a subject’s typing sessions, is randomly split to a training *I*′_*a*,*TRN*_ (80%) and a testing *I*′_*a*,*TST*_ subset (20%), *a*∈{*HT*, *NFT*, *NP*}. Training subsets *I*′_*a*,*TRN*_ are subjected to a recursive feature elimination procedure that updates feature vectors by selecting the most discriminant features. An upper limit of five selected features is set to avoid the “curse of dimensionality”^34^. The updated *I*′_*a*,*TRN*_ serve to train each of the first-stage classifiers. For each classifier, grid search is performed at first, using the updated *I*′_*a*,*TRN*_ and an inner 4-fold cross-validation for hyper parameter optimization. The outcomes of this process are three optimised models in terms of discriminant features and classifier parameters. Following this, the testing subsets *I*′_*a*,*TST*_ are used to test the optimised models. Testing probabilities *P*_*a*_, *a*∈{*HT*, *NFT*, *NP*}, outputted from the three first-stage models, are afterwards grouped into three-element vectors *v*_*p*_ = [*P*_*HT*_, *P*_*NFT*_, *P*_*NP*_], which populate the training subset *I*′_*P*_ for the second-stage classifier. The latter is again optimised in terms of parameters based on the *I*′_*P*_ and a 4-fold cross-validation. The output of the optimised classifier is the final probability *P*_*f*_ that denotes whether a typing session belongs to a PD patient or a healthy control.

Once the training step completes, the left-out subject’s feature vector sets ${I^{\prime} }_{a}^{C}={I}_{a}\backslash {I^{\prime} }_{a}$, where *C* denotes the complement set and *a*∈{*HT*, *NFT*, *NP*}, are fed to the classification pipeline to test it under the optimised scenario (selected features and trained classifiers with optimised hyper parameters). The output of the pipeline is a set of prediction probabilities {*P*_*f*_} formed by classifying all typing sessions of the subject. Eventually, the left-out subject is classified as having PD or not by taking the mean of the set {*P*_*f*_}. The LOSO scheme is completed when all subjects are left out and afterwards evaluated against PD. For the first stage of the proposed methodology, combinations of four types of classifiers, i.e., Linear Support-Vector Machine^35^, Logistic Regression^36^, Random Forest^29^, and *k*-Nearest Neighbours, with three feature ranking-selection methods, i.e., Lasso, Ridge^28^, and Gini Impurity^37^, were examined in terms of classification performance. A Logistic Regression classifier was used in the second stage of the methodology, in all cases.

### Classification performance evaluation

Different classification pipeline configurations (different combinations of classifiers and feature selection methods) conceived here, as well as existing classification methods described in literature, are evaluated using the receiver operating characteristic (ROC) analysis. ROC analysis is an iterative process of varying the discrimination threshold of a binary classifier and outputting the (*Sensitivity*, *Specificity*) pair for each threshold. The ROC curve is then formed by plotting the output pairs of (1 − *Specificity*, *Sensitivity*). The analysis provides reliable insights into the performance of a classification model even when datasets are not completely balanced (45.5% healthy controls, 54.5% early PD patients in our case). To assess the statistical significance of classification results, sampling with replacement (1,000 bootstraps) is further used here to define a ROC curve distribution. The average value and the confidence intervals of the area under the ROC curve (AUC) over 1,000 bootstraps are used to evaluate the performance of each binary (PD patient vs. control) classification approach. Where reported, sensitivity/specificity values correspond to the optimal threshold for equal cost of misclassifying PD patients and healthy controls.

### Data Availability

All data generated and analysed during the current study are available from the corresponding author on a reasonable request.

## Electronic supplementary material

Supplementary Information
