# Supplementary material for: Touchscreen typing-pattern analysis for detecting fine motor skills decline in early-stage Parkinson’s disease
Source: Sci Rep. 2018 May 16;8:7663. doi: 10.1038/s41598-018-25999-0 (PMC5955899; doi:10.1038/s41598-018-25999-0)
Supplement: Supplementary file 1 — Supplementary Information [file 41598_2018_25999_MOESM1_ESM.docx]

**Supplementary Material for “Touchscreen typing-pattern analysis for detecting fine motor skills decline in early-stage Parkinson’s disease”**

**Dimitrios Iakovakis^1^, Stelios Hadjidimitriou^1^, Vasileios Charisis^1^, Sevasti Bostantzopoulou^2^, Zoi Katsarou^3^, and Leontios J. Hadjileontiadis^1,4^**

***^1^**Department of Electrical and Computer Engineering, Aristotle University of Thessaloniki, Thessaloniki, Greece.

**^2^**Third Neurological Clinic, G. Papanikolaou Hospital, Thessaloniki, Greece.

**^3^**Department of Neurology, Hippokration Hospital, Thessaloniki, Greece.

**^4^**Department of Electrical and Computer Engineering, Khalifa University of Science and Technology, Abu Dhabi, UAE.

*Correspondence and requests for materials should be addressed to L.H. (email: [leontios@auth.gr](mailto:leontios@auth.gr))

**ABSTRACT**

Parkinson’s disease (PD) is a degenerative movement disorder causing progressive disability that severely affects patients’ quality of life. While early treatment can produce significant benefits for patients, the mildness of many early signs combined with the lack of accessible high-frequency monitoring tools may delay clinical diagnosis. To meet this need, user interaction data from consumer technologies have recently been exploited towards unsupervised screening for PD symptoms in daily life. Similarly, this work proposes a method for detecting fine motor skills decline in early PD patients via analysis of patterns emerging from finger interaction with touchscreen smartphones during natural typing. Our approach relies on low-/higher-order statistical features of keystrokes timing and pressure variables, computed from short typing sessions. Features are fed into a two-stage multi-model classification pipeline that reaches a decision on the subject’s status (PD patient/control) by gradually fusing prediction probabilities obtained for individual typing sessions and keystroke variables. This method achieved an AUC=0.92 and 0.82/0.81 sensitivity/specificity (matched groups of 18 early PD patients/15 controls) with discriminant features plausibly correlating with clinical scores of relevant PD motor symptoms. These findings suggest an improvement over similar approaches, thereby constituting a further step towards unobtrusive early PD detection from routine activities.

| **Feature** | **PD patients average (avg.)** | **PD patients’ standard deviation (std.)** | **Controls avg.** | **Controls std.** | **Mann-Whitney**  **p-value** | |
| --- | --- | --- | --- | --- | --- | --- |
| **Normalized Pressure Features (aggregation per subject)** | | | | | | |
| $\bar{\mu_{i}}$ | 0.51 | 0.06 | 0.60 | 0.08 | p=0.002 | |
| $\sigma_{\mu_{i}}$ | 0.04 | 0.02 | 0.03 | 0.01 | p=0.046 | |
| $\bar{\sigma_{i}}$ | 0.11 | 0.01 | 0.11 | 0.01 | p=0.090 | |
| $\sigma_{\sigma_{i}}$ | 0.03 | 0.01 | 0.02 | 0.01 | p=0.300 | |
| $\bar{S_{i}}$ | -0.09 | 0.34 | -0.51 | 0.34 | p=0.002 | |
| $\sigma_{S_{i}}$ | 0.59 | 0.11 | 0.57 | 0.09 | p=0.200 | |
| $\bar{K_{i}}$ | -0.59 | 0.39 | -0.07 | 0.36 | p=0.000 | |
| $\sigma_{K_{i}},$ | 0.99 | 0.29 | 1.23 | 0.29 | p=0.016 | |
| $C_{U}$ | -0.12 | 0.64 | 0.03 | 0.37 | p=0.287 | |
| $\sigma_{c_{U}}$ | 3.25 | 1.90 | 2.41 | 2.54 | p=0.015 | |
| $\Sigma\left\vert C_{U} \right\vert]$ | 47.30 | 24.72 | 32.63 | 22.00 | p=0.005 | |
| **Hold Time Features (aggregation per subject)** | | | | | | |
| $\bar{\mu_{i}}$ | 0.16 | 0.06 | 0.09 | 0.03 | p=0.000 | |
| $\sigma_{\mu_{i}}$ | 0.02 | 0.01 | 0.01 | 0.00 | p=0.000 | |
| $\bar{\sigma_{i}}$ | 0.03 | 0.01 | 0.02 | 0.00 | p=0.000 | |
| $\sigma_{\sigma_{i}}$ | 0.02 | 0.01 | 0.00 | 0.00 | p=0.000 | |
| $\bar{S_{i}}$ | 0.29 | 0.21 | 0.14 | 0.12 | p=0.011 | |
| $\sigma_{S_{i}}$ | 0.70 | 0.11 | 0.63 | 0.10 | p=0.026 | |
| $\bar{K_{i}}$ | -0.14 | 0.27 | 0.06 | 0.35 | p=0.020 | |
| $\sigma_{K_{i}},$ | 1.10 | 0.39 | 1.20 | 0.38 | p=0.130 | |
| $C_{U}$ | -0.53 | 0.85 | 0.17 | 0.87 | p=0.020 | |
| $\sigma_{c_{U}}$ | 6.56 | 7.58 | 1.77 | 5.52 | p=0.002 | |
| $\Sigma\left\vert C_{U} \right\vert]$ | 67.58 | 84.74 | 12.23 | 38.06 | p=0.002 | |
| **Normalized Flight Time Features (aggregation per subject)** | | | | | | |
| $\bar{\mu_{i}}$ | - | - | - | - | - | |
| $\sigma_{\mu_{i}}$ | - | - | - | - | - | |
| $\bar{\sigma_{i}}$ | 0.03 | 0.01 | 0.02 | 0.00 | p=0.000 |  |
| $\sigma_{\sigma_{i}}$ | 0.02 | 0.02 | 0.00 | 0.00 | p=0.000 |  |
| $\bar{S_{i}}$ | 0.29 | 0.21 | 0.14 | 0.12 | p=0.011 |  |
| $\sigma_{S_{i}}$ | 0.70 | 0.11 | 0.63 | 0.01 | p=0.026 |  |
| $\bar{K_{i}}$ | -0.15 | 0.27 | 0.06 | 0.36 | p=0.020 |  |
| $\sigma_{K_{i}},$ | 1.01 | 0.40 | 1.20 | 0.38 | p=0.135 |  |
| $C_{U}$ | -0.44 | 0.70 | -0.36 | 0.58 | p=0.460 |  |
| $\sigma_{c_{U}}$ | 3.22 | 2.61 | 2.64 | 3.54 | p=0.060 |  |
| $\Sigma\left\vert C_{U} \right\vert]$ | 36.37 | 30.28 | 21.65 | 27.46 | p=0.026 |  |

**Table S.1.** Statistical features (1^st^ column) and their mean and standard deviation as calculated for the two groups (2^nd^ -5^th^ columns). The features were derived from aggregating features of the windowed signals per subject; hence, each subject’s typing sessions are represented by one feature vector. A two-sided Mann-Whitney U-test is performed for each feature to test the null hypothesis, i.e., that the two groups derive from the same distribution ($p$ values column). Total number of subjects n=33.

| **Feature** | **PD patients average (avg.)** | **PD patients’ standard deviation (std.)** | **Controls avg.** | **Controls std.** | **Mann-Whitney**  **p-value** |
| --- | --- | --- | --- | --- | --- |
| **Normalised Pressure Features (aggregation per session)** | | | | | |
| $\bar{\mu_{i}}$ | 0.51 | 0.06 | 0.60 | 0.08 | p=0.000 |
| $\sigma_{\mu_{i}}$ | 0.03 | 0.02 | 0.02 | 0.01 | p=0.000 |
| $\bar{\sigma_{i}}$ | 0.11 | 0.02 | 0.11 | 0.02 | p=0.013 |
| $\sigma_{\sigma_{i}}$ | 0.02 | 0.01 | 0.02 | 0.01 | p=0.040 |
| $\bar{S_{i}}$ | -0.11 | 0.42 | -0.49 | 0.44 | p=0.000 |
| $\sigma_{S_{i}}$ | 0.52 | 0.21 | 0.42 | 0.23 | p=0.000 |
| $\bar{K_{i}}$ | -0.54 | 0.61 | -0.12 | 0.71 | p=0.000 |
| $\sigma_{K_{i}},$ | 0.80 | 0.50 | 0.87 | 0.59 | p=0.247 |
| $C_{U}$ | -0.56 | 43.21 | 8.81 | 60.69 | p=0.468 |
| $\sigma_{c_{U}}$ | 191.70 | 171.56 | 290.93 | 399.54 | p=0.004 |
| $\Sigma\left\vert C_{U} \right\vert]$ | 2749.36 | 2785.02 | 4881.83 | 5006.93 | p=0.000 |
| **Hold Time Features (aggregation per session)** | | | | | |
| $\bar{\mu_{i}}$ | 0.16 | 0.06 | 0.10 | 0.03 | p=0.000 |
| $\sigma_{\mu_{i}}$ | 0.01 | 0.01 | 0.01 | 0.00 | p=0.000 |
| $\bar{\sigma_{i}}$ | 0.03 | 0.01 | 0.02 | 0.00 | p=0.000 |
| $\sigma_{\sigma_{i}}$ | 0.01 | 0.01 | 0.00 | 0.00 | p=0.000 |
| $\bar{S_{i}}$ | 0.25 | 0.36 | 0.13 | 0.32 | p=0.010 |
| $\sigma_{S_{i}}$ | 0.58 | 0.27 | 0.48 | 0.26 | p=0.000 |
| $\bar{K_{i}}$ | -0.12 | 0.63 | 0.04 | 0.70 | p=0.028 |
| $\sigma_{K_{i}},$ | 0.86 | 0.61 | 0.82 | 0.63 | p=0.165 |
| $C_{U}$ | -0.65 | 6.11 | 0.32 | 4.20 | p=0.000 |
| $\sigma_{c_{U}}$ | 12.20 | 37.90 | 2.74 | 27.82 | p=0.000 |
| $\Sigma\left\vert C_{U} \right\vert]$ | 90.00 | 271.20 | 18.60 | 188.70 | p=0.000 |
| **Normalized Flight Time Features (aggregation per session)** | | | | | |
| $\bar{\mu_{i}}$ | - | - | - | - | - |
| $\sigma_{\mu_{i}}$ | - | - | - | - | - |
| $\bar{\sigma_{i}}$ | 0.43 | 0.13 | 0.34 | 0.13 | p=0.000 |
| $\sigma_{\sigma_{i}}$ | 0.12 | 0.05 | 0.09 | 0.05 | p=0.000 |
| $\bar{S_{i}}$ | 0.90 | 0.53 | 1.51 | 0.61 | p=0.000 |
| $\sigma_{S_{i}}$ | 0.56 | 0.22 | 0.57 | 0.36 | p=0.136 |
| $\bar{K_{i}}$ | 0.66 | 1.43 | 2.64 | 2.61 | p=0.000 |
| $\sigma_{K_{i}},$ | 1.31 | 0.91 | 2.07 | 1.50 | p=0.000 |
| $C_{U}$ | 0.00 | 0.04 | 0.00 | 0.04 | p=0.248 |
| $\sigma_{c_{U}}$ | 0.22 | 0.21 | 0.16 | 0.16 | p=0.002 |
| $\Sigma\left\vert C_{U} \right\vert]$ | 2.79 | 2.79 | 1.84 | 2.06 | p=0.000 |

**Table S.2.** Statistical features (1^st^ column) extracted for each typing session with the corresponding average and standard deviation values for the two populations (2^nd^- 5^th^ columns). A two-sided Mann-Whitney U-test is performed to test the null hypothesis, i.e., that the sessions' features derive from the same distribution ($p$ values column). Total number of sessions n=275.

**Information**

**Source**

**Diagnostic**

**Metric**

|  | **Single Metrics** | | | **Combined**  ***P_f_*** |
| --- | --- | --- | --- | --- |
|  | ***P_HT_*** | ***P_NFT_*** | ***P_NP_*** |  |
| Area Under Curve, 95% CI | 82.1, [69.1, 92.8] (%) | 79.0, [64.9, 90.7] (%) | 67.0, [51, 82.0] (%) | 92.0, [82.0, 98.0] (%) |
| Sensitivity, 95% CI | 70.0, [45.7, 88.1] (%) | 59.1, [36.3, 79.3] (%) | 61.1, [35.7, 82.7] (%) | 82.0, [52.9, 95.7] (%) |
| Specificity, 95% CI | 92.3, [64.0, 99.8] (%) | 81.8, [48.2, 97.7] (%) | 73.3, [44.9, 92.2] (%) | 81.0, [58.6%, 96.4] (%) |
| Positive Predictive Value, 95% CI | 93.3, [67.1, 98.9] (%) | 86.7, [63.9, 96.0] (%) | 73.3, [52.4, 87.3] (%) | 80.00, [58.0, 92.1] (%) |
| Negative Predictive Value, 95% CI | 66.7, [50.1, 79.9] (%) | 50.0 [36.0, 64.0] (%) | 61.1, [44.9, 75.1] (%) | 83.3, [64.0, 93.3] (%) |
| Diagnostic Accuracy | 78.8, [61.1, 91.0] (%) | 66.7 [48.2, 82.0] (%) | 66.7, [48.2, 82.0] (%) | 81.8, [64.5, 93.0] (%) |

**Table S.3.** Diagnostic metrics of prediction probabilities, i.e., $\{P_{HT}\},$ {$P_{NFT}\}$, {$P_{NP}\}$, estimated independently from features of single keystroke variable, as well as of ${\{P}_{f}\}$, i.e., the fused outcome of the latter probabilities sets as derived by the proposed two-stage classification pipeline. Mean-voting was applied in all cases as the final step to obtain a single prediction probability on the subject's condition (PD patient or control). Thresholds were computed as the closest-to-(0,1) cut-off points of the corresponding bootstrapped ROC curves, for equal cost of misclassifying patients and controls. CI: Confidence Interval.

| **NFT features** | **# Selected during LOSO validation** |
| --- | --- |
| $\bar{S_{i}}$ | 12 |
| $\sigma_{S_{i}}$ | 29 |
| $\bar{K_{i}}$ | 33 |
| $\sigma_{K_{i}},$ | 33 |
| $C_{U}$ | 8 |
| $\sigma_{c_{U}}$ | 14 |
| $\Sigma\left\vert C_{U} \right\vert]$ | 3 |

**Table S.4.** Selection frequency of NFT features of the Arroyo-Gallego et al.’s^21^ extracted on our dataset and subjected to LOSO validation process**.**


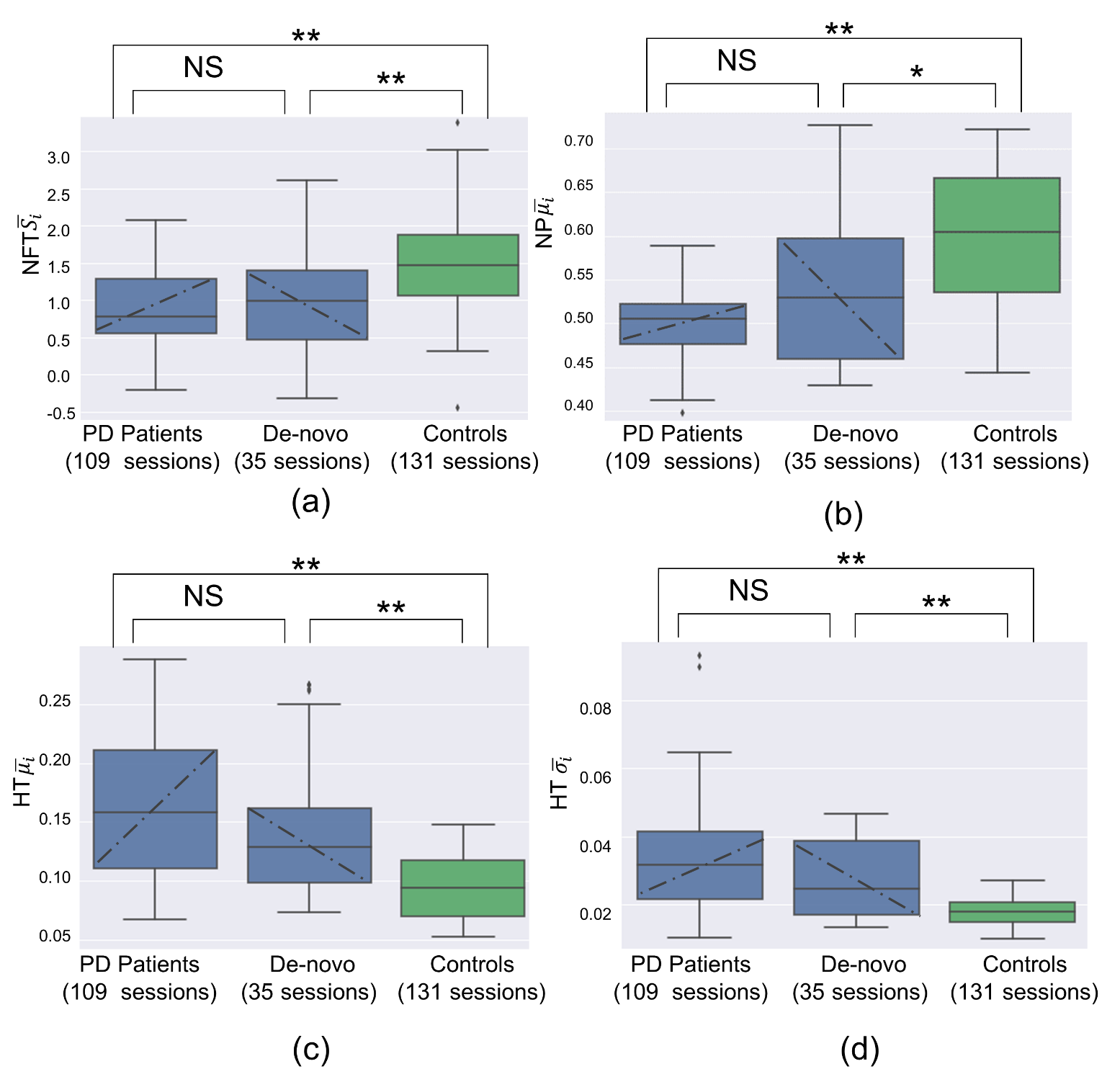


**Figure S.1.** Comparison of the distribution of (a) $NFT \bar{S}_{i}$, (b) $NP \bar{\mu}_{i}$, (c)$HT \bar{\mu}_{i}$, and (d) $HT \bar{\sigma}_{i}$ features, computed over all typing sessions of controls vs. PD patients under medication (PD patients) vs. De-novo PD patients (recently diagnosed with PD and never taken PD medication). *: Statistically significant difference at p<0.05; **: Statistically significant difference at p<0.001; NS: non-statistically significant difference.
